# The Association Between Oral Helicobacter pylori and Gastric Complications: A Comprehensive Review

**DOI:** 10.7759/cureus.24703

**Published:** 2022-05-03

**Authors:** Njoud K Alkhaldi, Waad K Alghamdi, Maryam H Alharbi, Albandri S Almutairi, Faisal T Alghamdi

**Affiliations:** 1 General Medicine and Surgery, College of Medicine, Princess Nourah bint Abdulrahman University, Riyadh, SAU; 2 Department of Oral Biology, Faculty of Dentistry, King Abdulaziz University, Jeddah, SAU

**Keywords:** review, oral cavity, helicobacter pylori, gastric complications, gastric infection, association

## Abstract

*Helicobacter pylori* (*H. pylori*) is linked to chronic gastritis, duodenal or gastric ulcers, and gastric cancer (GC). Because the oral cavity is the first component of the gastrointestinal tract (GIT) and the entrance point for *H. pylori*, it has been proposed as a possible reservoir of *H. pylori*. As a result, a putative oral-oral transmission pathway of *H. pylori* poses the possibility of whether personal contact, such as kissing or sharing a meal, might trigger *H. pylori* transmission. As a result, several investigations have been done on this issue using various approaches for detecting *H. pylori* in oral and stomach samples. Furthermore, the relationship between *H. pylori* and gastrointestinal disorders has yet to be studied. The evidence for the association between *H. pylori* and gastric diseases and their complications is still a controversial subject due to the existing literature in this review. The goal of this comprehensive review was to collect all available published articles and critically evaluate existing investigations looking into the relationship between oral *H. pylori* contamination and the danger of gastric complications. Few studies indicated an association between *H. pylori* and gastric diseases. Furthermore, more longitudinal randomized clinical studies to further investigate the association between *H. pylori* and gastric diseases are warranted.

## Introduction and background

*Helicobacter pylori *(*H. pylori*) is a major agent that accounts for 62% of gastric complications among Pakistanis. It causes chronic gastritis and peptic and duodenal ulcers [1]. The risk of developing *H. pylori* infection is linked directly to multiple microorganisms, host genetic, and environmental factors, as well as attachment and colonization, and the severity of gastritis depends on the *H. pylori* agent. Furthermore, neutrophil and mononuclear cell diffusion in the gastrointestinal (GI) lining results in chronic active gastritis. The oral cavity, which includes the oral mucosa, saliva, and periodontal spaces, is where *H. pylori* are found [2]. *Helicobacter pylori* infection is strongly associated with the development of oral diseases, such as periodontitis, which is an infection of the gum manifested as an increase in pocket depth and clinical attachment loss [3]. Of the more than 300 bacterial species linked to deep periodontal pockets and severe periodontitis, spirochetes and Gram-negative rods are the most common [4].

*Helicobacter pylori *survival calls for an acidic pH (1.4) withinside the stomach, which favors the development of *H. pylori* and allows it to create enough amount of the enzyme urease (Ure), which converts UreA to carbon dioxide and ammonia. For colonization in the gastrointestinal mucosa, all *H. pylori* strains produce more urease enzymes than other pathogens. Urease, which is encoded by a set of genes comprising UreA, UreG, UreE, UreI, UreB, UreH, and UreF, is a key enzyme in *H. pylori* pathogenesis [5,6]. The presence of dental biofilm in chronic gastritis can lead to *H. pylori* colonization. The eradication rate of *H. pylori* in the stomach is lower in patients who tested positive for oral *H. pylori* than in those who tested negative for oral *H. pylori* [7]. Similar findings have been observed in India [8].

The presence of adhesive molecules is conditioned for *H. pylori* colonization and determination. *Helicobacter pylori* have a wide spectrum of adhesive capabilities; it is thought to be able to bond with a wide range of extraordinary carbohydrates, including the binding adhesins babA, sabA, Leb, and sLex [9]. Furthermore, according to a recent study, cagA-positive strains are linked to stomach illness [10]. The purpose of cagA is to interact with the host signaling molecule called tyrosine phosphatase. It is a result of structural changes in the gastrointestinal mucosa [11]. VacA creates a special strain of *H. pylori*; this strain plays a key role in the development of gastric disorders, and vacA produces around 50% of it [12]. A dynamic poison of 87-94 kDa, an N-terminal domain (usually responsible for vacA activation), and a C-terminal region make up vacA initiator, a 140 kDa macromolecule (liable to bind the protein to host cells) [13].

In patients who have been infected with *H. pylori*, there is a direct link between the ulcer and the growth of stomach cancer [14]. Oral hygiene and oral health condition are directly associated with oral *H. pylori* infection, and the incidence of infection can be reduced by oral hygiene and periodontal health status [15,16]. Studies from Brazil [16] and Venezuela [17] have neglected to build a relationship between the impacts of oral *H. pylori* and gastric *H. pylori *complications. There have been no previous reviews that investigated the association between oral *H. pylori* and gastric diseases. Consequently, the goal of this comprehensive review is to collect all available published articles and critically evaluate existing investigations looking into the relationship between oral* H. pylori* contamination and the danger of gastric complications. Nonetheless, the proposed theory is that oral *H. pylori* is associated with gastric diseases.

## Review

Material and methods

By evaluating relevant literature, the current comprehensive review intends to research the relationship between oral *H. pylori* contamination and the danger of gastric complications. Between June and December 2021, a search was conducted in PubMed, Scopus, Web of Science, and Google Scholar databases. Initially, papers written in the English language were considered. In the search, the following keywords were used: “*Helicobacter pylori*,” “oral infection,” “gastric cancer,” “gastric infection,” “oral complications,” “oral complications,” and “gastric complications.” All the available review and original research papers deemed relevant to this topic were considered and thoroughly investigated. No time restrictions were used in this review. All of the selected papers’ reference lists were then hand-searched for additional papers judged relevant by all authors. The selection process resulted in the selection of 752 papers for a comprehensive evaluation, resulting in the final selection of 46 papers in this review that met the inclusion criteria.

Prevalence of *H. pylori*

The presence of adhesive molecules is conditioned for *H. pylori* colonization and determination. *Helicobacter pylori *have a wide spectrum of adhesive capabilities; it is thought to be able to bond with a wide range of extraordinary carbohydrates, including the binding adhesins babA, sabA, Leb, and sLex [9]. Moreover, a recent report concluded that cagA-positive strains are suggested to have an association with gastric disease [10]. CagA interacts with tyrosine phosphatase, a host signaling molecule that is a result of structural changes in the GI mucosa [11]. VacA creates a particular *H. pylori* strain, which plays a significant role in the development of gastrointestinal disorders, of which vacA produces roughly 50% [12]. A dynamic toxin of 87-94 kDa, an N-terminal domain (normally accountable for vacA activation), and a C-terminal location make up vacA initiator, a 140 kDa protein (vulnerable to bind the protein to host cells) [13].

In patients who have been infected with *H. pylori*, there is a direct link between the ulcer and the growth of stomach cancer [14]. The oral hygiene condition has a direct association with oral *H. pylori* infection, and the incidence of infection can be reduced by oral hygiene condition and periodontal status [13,15]. Studies from Brazil [16] and Venezuela [17] have failed to construct a relationship between the influences of oral *H. pylori* and the danger of gastric *H. pylori *problems. As a result, the purpose of this complete evaluation is to examine the link between oral *H. pylori* infection and the hazard of gastrointestinal complications. Regardless, the proposed theory is that oral *H. pylori* contamination is related to gastrointestinal infection.

*Helicobacter pylori* infection is one of the most common chronic bacterial infections globally, with prevalence varying according to each country’s economic growth. The organism is passed down from generation to generation and varies from person to person, and some studies believe that the mouth cavity is a major reservoir for *H. pylori*, besides the stomach and intestines [18]. Although the mouth may be a source of transmission, it is uncertain whether the mouth is a common source of stomach reinfection following therapy [19]. Through this comprehensive review article, it was clear that there is an association between oral *H. pylori *and gastric diseases.

*Helicobacter pylori* is a Gram-negative, microaerophilic bacterium that is commonly found in the stomach. It is considered one of the risk factors for a variety of digestive clinical presentations, including ulcers, chronic active gastritis, peptic ulcer, and, much less commonly, stomach cancer. Urease UreA, UreC, vacA, babA, and cagA are the virulence factors most commonly linked with the clinical isolates of *H. pylori* in individuals diagnosed with peptic ulcer or malignant illness [20]. Furthermore, the most essential virulence factors for the raised danger of gastrointestinal problems by *H. pylori* have been identified as cagA, vacA, and babA2 [21], in addition to factors such as age at infection, *H. pylori* strains, genetics, phenotypes, and the response of the host to the secretion capacity of gastric acid/mucosa [22].

The prevalence of *H. pylori* was reported in different articles. Momtaz et al. [23] reported that the prevalence of *H. pylori* was 90.74% among patients with peptic ulcer disease (PUD), 80% among patients with gastric cancer (GC), and 74.13% among patients with non-ulcer dyspepsia (NUD) by polymerase chain reaction (PCR) assay according to a cross-sectional descriptive study conducted on 300 samples including antral gastric, saliva, dental plaque, and stool samples all obtained from patients who underwent upper gastrointestinal tract (GIT) endoscopy. On the other hand, Vaziri et al. [24] found that *H. pylori *were isolated from individuals with a variety of gastroduodenal diseases, including 71 isolated from 177 patients. The majority of the infected patients had chronic gastritis, whereas the remaining patients had duodenitis, intestinal metaplasia, hyperplasia, and gastric cancer disorders [24], accounting for 84.6%, 9.8%, 2.8%, 1.4%, and 1.4%, respectively. Regarding using the fast urease test or Giemsa staining, Cai et al. [25] discovered that 46 of 235 patients tested positive for gastric *H. pylori* infection. In oral and stomach samples, 26 individuals out of 46 had amplified *H. pylori* 16S rDNA. In oral samples, 12 (46.1%) individuals tested positive for the* H. pylori* cagA gene, which is lower than that found in the gastric mucosa [25]. PCR was used to detect *H. pylori* infection in stomach biopsies and oral samples in the study of Ansari et al. [20]; there were 247 *H. pylori*-positive subjects and 320 *H. pylori*-negative subjects. According to the study by Zhao et al. [26], 35 out of 80 patients with chronic gastritis had cagA-positive *H. pylori *infection, 32 patients had *H. pylori*-negative infection, and 13 patients had cagA-negative *H. pylori* infection. Gastric mucosa and tongue covering specimens were collected [26]. The study by Wu et al. [27] included 38 subjects, 27 of whom had superficial gastritis (SG) and 11 of whom had stomach cancer. In the gastric cancer (GC) group, there were five *H. pylori-*negative subjects and six *H. pylori*-positive subjects, while in the SC group, there were seven *H. pylori*-negative subjects and 20 *H. pylori*-positive subjects [27].

Another study confirmed that 164 out of 200 gastric biopsy specimens, which accounts for 82%, have been confirmed to have gastric *H. pylori* contamination via the RUT and UreC gene [28]. In this study, out of the 164 *H. pylori*-positive samples, a collection of 151 (92.07%) have been cagA-positive [28]. It additionally confirmed that 16 patients had gastric ulcers, three had duodenitis, three had gastric cancer, 22 had duodenal ulcers, 34 had gastric nodularity, 52 had gastric erosion, and 159 had gastritis [28]. It corresponds to the following percentages: 9.76%, 1.83%, 1.83%, 13.41%, 20.73%, 31.71%, and 96.95%, respectively. In addition, a study was conducted in United Arab Emirates that included 350 subjects who were tested for *H. pylori *using the stool antigen test [29]. The results showed that the prevalence of *H. pylori* infection was considered 41%, including both children and adults [29]. Another study carried out in India showed that 62% (329/530) of patients screened were *H. pylori*-infected [30]. They were investigated for *H. pylori* infection through histopathological examination and rapid urease test of biopsy specimen after being subjected to upper gastrointestinal endoscopy [30]. All these studies show a high prevalence of *H. pylori* infection. Thus, our review confirmed that there is a high prevalence of *H. pylori* infection in association with gastric diseases.

Association between *H. pylori* and gastric diseases and their complications

Different studies discussed the association between *H. pylori* and gastric diseases and their complications [20-27]. Children’s oral cavities include separate *H. pylori *strains, in keeping with Cai et al. [25], but a minimal gene homology between gastric and oral strains in kids with persistent gastritis was found. *Helicobacter pylori *is spiral and a Gram-negative microaerobic bacterium thought to cause chronic gastritis, peptic ulcer disease, GC, and gastric mucosal-related malignant lymphoma, and this will help speculate that the gastrointestinal tract includes an important reservoir of *H. pylori*, which is the oral cavity [25]. Momtaz et al. [23] released an article that suggested a link between gastric cancer and vacA status, similar to previous studies from Iran [2,11]. However, statistically, no link between gastric cancer and vacA status was observed (p = 0.100) [2,11]. Also, the prevalence of *H. pylori* was 90.47% among patients with PUD, 80% among patients with gastric cancer, and 74.13% among patients with NUD by PCR assay [23]. An article published by Wu et al. [27] comparing superficial gastritis (SG) patients and GC patients positive with *H. pylori* showed that the core shared oral bacteria in the gastric mucosa of gastric cancer patients was greatly influenced by* H. pylori* infection in terms of prevalence and relative abundance. We also found no link between *H. pylori* infection and the tongue coating microbiome composition. This could mean that *H. pylori* cause gastric dysbiosis by interfering with the ability of oral bacteria to colonize the gastric mucosa. On the other hand, the abundance of other bacterial taxa in a GC-specific microbiota shows that non-*H. pylori *gastric microorganisms may also play a role in gastric carcinogenesis. *Fusobacterium* spp. (66.2%), *Helicobacter* spp. (65.4%), *Rudaea* spp. (61.6%), and *Porphyromonas* spp. (53.3%) were the most commonly shared in GC patient samples, while *Fusobacterium* spp. (72.7%), *Porphyromonas* spp. (67.8%), *Rudaea* spp. (65.8%), and *Leptotrichia* spp. (63.5%) were the most commonly shared in SG patient samples [27]. The findings of the study by Ansari et al. [20] documented that cagA, vacA, and babA2 are the most important virulence factors that increase the risk of gastric complications due to *H. pylori*; the odds of developing gastroesophageal reflux grade II (OR = 1.458, 95% CI = 0.659-3.226), normal upper GIT mucosa with lax esophageal sphincters (OR = 1.215, 95% CI = 0.285-5.181), and duodenal ulcer/duodenitis (OR = 2.187, 95% CI = 0.225-21.278) are increased by oral exposure to *H. pylori*. This shows that *H. pylori* infection begins in the oral cavity. In both patients and controls, there was no significant connection (p > 0.05) between oral *H. pylori* and stomach infections/complications. The article by Vaziri et al. [24], on the other hand, showed that vacA s1 and m1 genotypes have been linked to *H. pylori*-related disorders; however, due to their low or non-vacuolating activities, the vacA s2 and m2 strains are rarely linked to peptic ulcers and stomach cancer [24]. Yamaoka et al. [31] reviewed that the vacA m1 genotype is dominant in East Asia and its southern areas, where the incidence of gastric cancer is high in the northern areas of East Asia and low in its southern areas. CagA (p = 0.011, p = 0.020) and babA gene-carrying strains (p = 0.031) were found to have significant correlations with severe active chronic gastritis. In the combined genotypes, only cagA+/vacAs1m1/iceA2/babA+ genotype showed correlation with severe active chronic gastritis (p = 0.025) [24]. According to the study of Zhao et al. [26], the development of a variety of illnesses is due to the unregulated lipopolysaccharide (LPS) production by the infection. Infection with cagA-positive *H. pylori* strains is raised. In the gastric mucosa, LPS can restrict *H. pylori* elimination by reducing the inflammatory response and the progression of chronic gastritis [32]. Furthermore, LPS exposure has been linked to a number of disorders, including endotoxemia, allergy, and autoimmune disease [33], obesity [34], and neurosystemic disorders (including autism [35] and Alzheimer’s disease [36]). With the use of linear discriminant analysis effect size (LEfSe), a statistically significant determination of KEGG pathways for each group was proved. The gastric microbiome of *H. pylori*+/cagA+ patients, in comparison to the *H. pylori* group, was significantly enriched for several pathways. Those pathways were involved in bacterial motility proteins, secretion system, lipopolysaccharide biosynthesis proteins, lipopolysaccharide biosynthesis, flagellar assembly, oxidative phosphorylation, and ribosome. In addition, in the *H. pylori*+/cagA− group, the secretion system and LPS biosynthesis proteins were also upregulated. Furthermore, there is no significant difference in functions between both groups. *Helicobacter pylori* infection induces changes in the function of gastric microbiota. There is a significant difference in the abundance of KEGG pathways between both groups identified by LEfSe. This is between *H. pylori-*positive/cagA-positive and *H. pylori*-negative patients, and between *H. pylori-*positive/cagA-positive and *H. pylori*-positive/cagA-negative patients. For the factorial Kruskal-Wallis test (α value) and discriminative features, they are set at p = 0.01 and p = 3.00, respectively, on log-linear discriminant analysis [26].

The oral cavity has been discussed in previous systematic review studies as a potential reservoir of *H. pylori* and might play a role in GI infections and complications since it is the entry port and the first component of the GI system. According to a previous study, more than half of the world’s population is infected with *H. pylori* [37]. However, no report has discussed the prevalence of *H. pylori* infection in the whole GIT. To the best of our knowledge, various reviews related to this topic were published, but the relation between oral colonization and *H. pylori* and gastric colonization, infection, and/or complications is still debatable. According to two review articles published in 2014, there was no obvious association between oral and gastric *H. pylori* [25,38], and the oral cavity cannot be a reservoir for gastric *H. pylori* infections, adding to the fact that different strain was found for oral *H. pylori* in their pediatric sample. Furthermore, a review study was published in 2021 supporting that the oral cavity can be “contaminated” with *H. pylori* by consuming *H. pylori*-infected food, and it criticized some previously published papers by justifying that there is no definitive evidence that *H. pylori* was ever been isolated from the oral cavity in the studies that have been considered in this review, adding to the fact that the influence of gastroesophageal reflux on the oral cavity has not been investigated [39]. *Helicobacter pylori* have been linked to some gastric complications, including chronic gastritis, peptic ulcer, hemorrhage, gastric perforation, cancer, mucosa-associated lymphoid tissue (MALT), lymphoma, and gastric outlet obstruction. There is an association between oral and gastric *H. pylori* [25,38]. Although the oral cavity is the entry port of the GI system and might be colonized with the infection, complications were not reported.

There is strong evidence that the occurrence of gastritis is greatly associated with *H. pylori* bacterial infection. According to the study by Kilmartin [40], infections of the stomach with *H. pylori* are common worldwide and can lead to major medical problems such as gastritis and associated complications, as well as gastric cancer or lymphoma.

The Japanese study by Miyabayashi et al. [41] conducted on 47 patients with *H. pylori* gastritis checked out the connection between gastric eradication fulfillment and *H. pylori* incidence withinside the oral cavity, as measured using nested PCR before and after eradication medication (the specificity PCR amplification process is improved by nested PCR). According to the findings, *H. pylori* in the mouth affects the outcome of eradication therapy and is associated with the recurrence of stomach infections. We urge that nested PCR be used to test oral *H. pylori* and that, if positive, it be regarded as a causative factor in refractory or recurring cases. The results showed that at four weeks after medication, the eradication success rate in oral *H. pylori*-positive cases (12/23, 52.1%) was substantially lower than in *H. pylori*-negative cases (22/24, 91.6%) (p = 0.0028). Out of 23 oral *H. pylori*-positive cases, only 16 (69.5%) were disease-free two years later, compared to 23 of the 24 oral *H. pylori*-negative cases (95.8%) (p = 0.018) [41].

The study of Malaty et al. [42] found the presence of *H. pylori* in dental plaque. This means that dental employees may be more susceptible to *H. pylori* infection as a result of their work. In the cross-sectional survey of 239 dental employees conducted in 37 Texas localities, the presence of IgG antibodies to *H. pylori* was used to determine *H. pylori* infection using a specific and sensitive enzyme-linked immunoassay (ELISA) [42]. *Helicobacter pylori* infection was found in 24% of dental professionals, including 17% of dentists, 18% of dental hygienists, 34% of dental assistants, and 25% of dental students. With age, the prevalence increased considerably (p = 0.05). Non-whites had a considerably greater rate of *H. pylori* infection (29/63 (46%)) than whites (29/176 (16%)) (p = 0.001) [37].

Marshall was the first to suggest a link between *H. pylori* and active chronic gastritis, duodenal ulcer, and gastric ulcer when he ingested this bacteria; he was awarded the Nobel Prize in 2005 with Robin Warren in a self-experiment [43].

In 100 consecutive consenting patients presenting for gastroscopy, biopsies were collected from the intact regions of the antral mucosa. In 58 patient samples, spiral or curved bacilli were found. Bacilli cultivated from 11 of these biopsies were Gram-negative, flagellate, and microaerophilic, indicating that they were a new *Campylobacter* spp. [44]. The bacteria were found in nearly all individuals with active chronic gastritis, duodenal ulcers, or stomach ulcers, suggesting that they may play a role in the genesis of these conditions. The study concluded that the most common signs of *H. pylori* gastritis are gastric mucosal damage and duodenal erosion. In this study, rats infected with both *Streptococcus mutans* and *H. pylori* experienced more damage to their digestive tracts than rats that were not infected with *H. pylori*. These rats, on the other hand, did not show signs of severe gastrointestinal disorders such as gastric cancer [44].

The study of Smoot [45] demonstrated that an extensive inflammatory response and stomach epithelial cell damage characterized *H. pylori*-associated gastritis. The findings revealed that *H. pylori* can cause direct damage to gastric epithelial cells by secreting enzymes and producing toxins. Adherence is likely to be an efficient route for delivering toxins to host cells, and intimate contact may cause stomach epithelial cell damage in and of itself. Cell damage occurs when cells are exposed to bacteria or soluble bacterial proteins, resulting in growth inhibition and possibly cell death [45].

The evidence of the association between *H. pylori* and gastric diseases and their complications is still a controversial subject due to the existing literature in this review. Few studies indicated that there is an association between *H. pylori* and gastric diseases. This narrative review of the literature has some limitations. There were few studies included in this review. Also, only 11 (23.91%) of the 46 references used were published in the last five years.

Treatment

The standard management for *H. pylori* infection if the results come positive is a sequential therapy for* H. pylori *eradication for 10 days starting with five days of one antibiotic, usually amoxicillin and proton pump inhibitor, followed by five days of proton pump inhibitors (esomeprazole, lansoprazole, omeprazole, or pantoprazole) and two antibiotics, which are usually clarithromycin and amoxicillin. Other types of antibiotics can be used in the last five days (amoxicillin and metronidazole or clarithromycin and metronidazole). However, if the patient’s results came negative, medications such as H2 receptor antagonists (ranitidine or famotidine) and proton pump inhibitors are administered [46]. On top of that, *H. pylori* patients should pay close attention to routine visits to the dentist to clean the dental calculus, as the dental calculus serves as a reservoir for this germ and can cause reinfection even after being treated with antibiotics.

## Conclusions

It can be concluded that this review showed that the association between oral *H. pylori* and gastric complications is still a controversial subject. Few studies showed that *H. pylori* species are related to or cause a curable chronic infection and their complications. Although this infection may be difficult to eradicate in some patients and the chance of recurrence is high, it is treatable with triple therapy antibiotics. Reduction of the chance of recurrence can be maintained by oral hygiene and disease management. Other common conditions, such as *H. pylori* infection, can be improved by recognizing and treating an associated oral infection. At the same time, it remains to be seen whether these general and oral conditions are the result of some common etiological problem or stress-like factors. Furthermore, more longitudinal, randomized clinical studies to further investigate the association between *H. pylori* and gastric diseases are warranted.
